# Improvement of nursing students' learning outcomes through scenario-based skills training

**DOI:** 10.1590/1518-8345.1310.2790

**Published:** 2016-08-08

**Authors:** Nurcan Uysal

**Affiliations:** 1 PhD, Assistant Professor, Nursing Department, Faculty of Health Sciences, Gediz University, Seyrek, İzmir, Turkey.

**Keywords:** Nursing, Students, Learning, Nursing Research

## Abstract

**Objective:**

this study analyzed the influence of scenario-based skills training on students' learning skills.

**Method:**

the author evaluated the nursing skills laboratory exam papers of 605 sophomores in nursing programs for seven years. The study determined the common mistakes of students and the laboratory work was designed in a scenario-based format. The effectiveness of this method was evaluated by assessing the number of errors the students committed and their achievement scores in laboratory examinations. This study presents the students' common mistakes in intramuscular and subcutaneous injection and their development of intravenous access skills, included in the nursing skills laboratory examination.

**Results:**

an analysis of the students' most common mistakes revealed that the most common was not following the principles of asepsis for all three skills (intramuscular, subcutaneous injection, intravenous access) in the first year of the scenario-based training. The students' exam achievement scores increased gradually, except in the fall semester of the academic year 2009-2010. The study found that the scenario-based skills training reduced students' common mistakes in examinations and enhanced their performance on exams.

**Conclusion:**

this method received a positive response from both students and instructors. The scenario-based training is available for use in addition to other skills training methods.

## Introduction 

Nursing education is a compilation of theory and practice components, and covers cognitive, affective, and psychomotor learning fields^1^. The nursing skills laboratory (NSL) is the most important field for psychomotor skills training^2^ ^-^ ^3^. The purpose of NSL work is to teach nursing students psychomotor skills and nursing care in a safe environment. The laboratory environment gives students the chance to "learn by practice". NSL is a controlled and safe learning environment^1^ ^,^ ^4^; it gives students the opportunity both to learn psychomotor skills and combine theory with practice, allowing them to experience self-learning and helping them to enhance their readiness for an actual clinical environment^5^ ^-^ ^6^. A variety of simulation methods are included in skills training during NSL. The use of simulations, mannequins and interactive videos, as well as critical thinking and making decisions by different techniques, such as role-playing, are the activities designed to demonstrate the skills and imitate an actual clinical environment^7^. 

Simulation training not only teaches skills to students at a desired level, but also provides a positive learning experience^4^. One study defined the primary training and assessment methods used in NSL as low to high-fidelity simulators, Standardized Patients (SPs), scenario-based simulation, Objective Structured Clinical Examination (OSCE) and Audio-Visual (AV) recording. OSCE is a performance-based examination where students are observed while demonstrating various clinical behaviors^8^. The use of OSCEs facilitates the assessment of psychomotor skills as well as knowledge and attitudes^9^. Some of the methods used in the instruction of basic nursing skills are the use of static mannequins, low-fidelity simulation (LFS), case studies and role- playing^10^. It is reported in the relevant literature that using low-fidelity simulation and non-complex scenarios has a positive effect on the instruction and assessment of nursing skills^11^ ^-^ ^12^. LFS and associated part-task training devices grant the user the opportunity to simulate an array of scenario based assessments and activities^12^. In one of the studies, LFS was considered as effective as high-fidelity simulation for technical skill acquisition, and both methods were significantly more effective than traditional didactic instruction^(13)^. The nursing school has been using static full-body mannequins as low-fidelity simulators in psychomotor skills training since its foundation (1994-1995). The assessment method used in the school is performance-based nursing skills laboratory examination (NSLE), which was adapted according to OSCE. OSCE determines to what extent students have reached the competencies defined and targeted in skills training^14^ ^-^ ^17^. Students are expected to put the skills they learned in lessons into precise practice in examinations. This practice is included in the third step of Miller's assessment pyramid, which is the "Shows How" step^15^ ^-^ ^16^. Students may not always show the skill precisely in NSLE, yet they have to perform the essential steps of the skill. This is important for students' being accepted as successful in NSLE and their competencies in the practice of skills being considered at an acceptable level before they start working at clinics. It is particularly important that invasive procedures are correctly applied to real patients in terms of patient safety. For this reason, it is a necessity in educational practice that students' competencies in learning nursing skills are continually increased. Thus, the scenario-based simulation method was added to NSL practices in the academic year 2007-2008. 

Learning theories indicate that students learn better when they practice the skills themselves in appropriate learning environments and participate in learning practices actively^18^. The best way to learn new things is to perform them and put them in practice^19^. A study was conducted that included three methods (scenario-based study groups with and without teacher, and simulation training) and found that scenario-based simulation training fits "learning by doing" the best^19^. According to the study developed with 12 health students; the study included four simulation scenarios and four short videos, and all students reported that they were satisfied with the training they were provided^20^. A study was conducted using the Laerdal SimMan Universal Patient Simulator with the purpose of determining the influence of realistic scenario-based simulation on nursing students' competence and confidence, and this method was considered effective^(21)^. The experimental group was provided with scenario-based training and the OSCE results were better than those of the control group. In a study students trained by means of a simulation that included vignette scenarios, OSCE and written examinations^4^. A study was conducted using static mannequins, scenarios, computer-aided directions and different active learning demonstrations, showing that students' achievement rates in case study questions ranged between 93% and 100% and that they also successfully reached the skill targets created for the program^11^. 

## Objective 

This study aimed to determine nursing students' common mistakes in NSLE and evaluate the effect of scenario-based NSL practices on reducing students' mistakes in exams and NSLE achievement scores. 

## Method 

This is a retrospective and quasi-experimental study. The author developed a retrospective analysis of the students' NSLE achievement scores in the fall semesters of the academic years 2005-2012. In the quasi-experimental part of the research, the results of scenario-based skills training are evaluated since the fall semester of 2007. The influence of the skills training on students' mistakes and NSLE achievement scores, which was provided in a scenario-based form starting from the fall semester of 2007, was evaluated through an analysis of exam papers between 2007 and 2012. The NSLE can be done in two stages: a short written exam (WE) and a performance exam in which students perform nursing skills under the supervision of an instructor, except for skills with a low difficulty level. Instructors assess each student's performance based on the control lists prepared specifically for each skill, and score them as "Done: 1 point" or "Not Done: 0 points". The NSLE success criterion is to get at least 70 out of 100 points.

The nursing school has been providing psychomotor skills training to students in NSL since its foundation (1994-1995). The instructors used mannequins and demonstration methods, such as a low-fidelity simulator in the laboratory. At the school of nursing, students and instructors provide their written and verbal feedback at the end of each semester for evaluation. The feedback indicated that the psychomotor skills training in the laboratory was focused on skills, and students and instructors began to regard it as a mechanical practice. According to the feedback provided by instructors who study with students in the skills laboratory and clinical practices, the students did not use critical thinking and problem-solving skills in the laboratory, did not want to spend too much time there, thought that the process was boring, and had problems in using their skills in the implementation area^22^. The NSL training process was re-structured for sophomore students in the academic year 2007-2008, due to the problems that arose in the laboratory practice process. 

### Procedure 

The researcher who delivered the second year laboratory practice course wrote scenarios for all the skills taught in the fall semester. The scenarios were inspired by the mistakes that students made frequently in NSLE exams and were based on problems that occur in actual clinical environments. An expert instructor nurse and the other instructors delivering the laboratory studies examined the scenarios created by the researcher. Then, the author revised the scenarios, taking the feedback of the experts into account. 

The author wrote specific study directions for each skill in order to achieve standardization among the small study groups in the laboratory. The author also held meetings with all the instructors who would deliver the laboratory courses a week before the laboratory studies. After each laboratory study, the author held meetings in order to receive feedback from the instructors. 

The author distributed the skill checklists to the students, along with the scenarios specifically written for each skill, such as printed materials and video, a week before the courses, and asked them to prepare before coming to the course. The laboratory study proceeded with the instructor showing how to perform the skill and students worked on it until they managed to perform it. Then, the students role-played the clinical scenario and were asked to offer solutions for the problem in that scenario. After the study was completed, the students filled out the NSL feedback forms and provided their verbal feedback. 

The author evaluated students' performance in the skills they learned in NSL using the NSLE, which was adapted to OSCE. NSLE results included both the written and performance examinations. First, the written examination was performed immediately after the performance examination. In the NSLE, there were control lists for each skill. Before the NSLE, the author held meetings with the instructors who would be in charge of the examination and informed them of the important points to pay attention to during the examination. 

### Sample

The sample of the study included the papers of the NSLE examination, which was administered to 605 students trained in the academic years 2005-2006 (n=60), 2006-2007 (n=72), 2007-2008 (n=82), 2008-2009 (n=64), 2009-2010 (n=90), 2010-2011 (n=105), and 2011-2012 (n=132). The papers of the NSLEs, which were administered in the spring semesters, were not included in the sample, since the spring curricula included skills that were not based on scenarios. 

The author obtained the written consent of the university's Non-Invasive Research Ethics Board (IRB: 86-GOA, 05.05.2011) and school administration. 

### Data collection and data analysis 

The exam control lists used for each skill in the NSLE was analyzed to produce the study data. The author examined all the NSLE control lists administered in the fall semesters between the years 2007 and 2012, and considered the skill steps marked as "Not Done" by the exam observers as mistakes. The author examined only scores on the NSLE control lists administered in the fall semesters between the years 2005 and 2006.

This study presents the students' common mistakes in intramuscular (IM) and subcutaneous (SC) injection, intravenous (IV) access skills included in the NSLE in the fall semesters between the years 2007 and 2012, as percentages and counts. The difference between the NSLE mean achievement scores in the fall semesters of the years 2005 and 2006, when scenario-based training was not provided, and those of the fall semesters between the years 2007 and 2012, when the scenario-based training was provided, was tested using one-way ANOVA. The study data was analyzed using the software package SPSS 22.0 (IBM Corporation). The significance level of the statistical analyses was p<0.05.

## Results

The total number of NSLE mistakes the students made in the fall semesters was as follows: 147 in 2007-2008 (n= 82); 118 in 2008-2009 (n=64); 185 in 2009-2010 (n=90); 107 in 2010-2011 (n=105); 106 in 2011-2012 (n=132). The study found that students' total mistakes in NSLE gradually dropped, except in the fall semester of the academic year2009-2010. The reason for this was that students neither filled out the rationale section in the control lists nor read the scenarios to solve the problem before coming to the NSL studies. Thus, students made more mistakes in NSLE. Table 1 present the percentages of the second year nursing students' mistakes in the practices of IM, SC injection, and IV access in the nursing skills laboratory exam.

**Table 1:** Distribution of the second year nursing students' mistakes in the practices of intramuscular and subcutaneous injection and intravenous access in nursing skills laboratory exam. İzmir, Turkey, 2007-2012

Nursing skills Intramuscular injection	2007 - 2008 year		2008 - 2009 year		2009 - 2010 year		2010 - 2011 year		2011 - 2012 year	
	n	%	n	%	n	%	n	%	n	%
Ignoring the asepsis principles	10	21.3	7	18.9	33	45.3	5	16.6	3	9.7
Not creating an airlock in the syringe	8	17	6	16.3	6	8.2	3	10	4	12.9
Making an incorrect decision for the injection site	4	8.5	3	8.1	6	8.2	2	6.6	3	9.7
Not telling the patient to take deep breaths	7	14.9	5	13.5	11	15.1	5	16.6	6	19.3
Not performing blood control before administering the medication	6	12.8	5	13.5	6	8.2	4	13.3	4	12.9
Neglecting privacy	5	10.6	4	10.8	3	4.1	3	10	4	12.9
Not making a record	5	10.6	4	10.8	5	6.8	3	10	3	9.7
Others (e.g. not informing the patient, moving the injector)	2	4.3	3	8.1	3	4.1	6	20.0	4	12.9
Total	47	100	37	100	73	100	30	100	31	100
Subcutaneous injection										
Ignore the asepsis principles	12	20.3	10	21.3	28	36.3	8	18.2	6	15.0
Not pull the drug in the right dose	14	23.8	12	25.6	18	23.4	9	20.6	7	17.5
Failure to identify the correct injection site	7	11.8	5	10.6	7	9.1	4	9.1	6	15.0
Not insert needle into SC tissue with right angle	9	15.2	7	14.9	7	9.1	6	13.6	5	12.5
Not inject the medicine into the SC tissue at the appropriate speed	6	10.3	5	10.6	7	9.1	5	11.4	6	15.0
Not making a record	7	11.8	5	10.6	6	7.8	4	9.1	4	10.0
Others (e.g. not informing the patient, moving the injector)	4	6.8	3	6.4	4	5.2	8	18.2	6	15.0
Total	59	100	47	100	77	100	44	100	40	100
Intravenous access										
Ignore the asepsis principles	10	24.4	8	23.5	15	42.8	7	21.2	6	17.2
Not insert needle into vein with right angle	5	12.2	4	11.7	3	8.6	4	12.1	4	11.4
Not inject the medicine into vein at the appropriate speed	6	14.6	5	14.7	3	8.6	5	15.2	6	17.2
Forget to untie the tourniquet	8	19.6	7	20.6	5	14.3	7	21.2	8	22.8
Not making a record	7	17.0	6	17.8	5	14.3	4	12.1	6	17.2
Others (e.g. not informing the patient)	5	12.2	4	11.7	4	11.4	6	18.2	5	14.3
Total	41	100	34	100	35	100	33	100	35	100

### The most common mistakes in IM injection

The distribution of students' mistakes in IM injection practice was as follows: the most common mistake in the first three years (2007-2009) was not following the principles of asepsis (consecutively 21.3%, 18.9% and 45.3%). It was determined that these mistakes were reduced in the fall semesters of 2010-2011 (16.6%) and 2011-2012 (9.7%). In the years 2010-2011 (16.6) and 2011-2012 (19.3%), the most common mistake was not telling the patient to take deep breaths during the injection.

### The most common mistakes in SC injection

The distribution of the most common mistakes by students during SC injection was: in the same order of the given years (20.3%, 21.3%, 36.3%, 18.2% and 15.0%), one of the most common mistakes was not following the principles of asepsis. Another was not taking a sufficient amount of medication into the injection, again in the same order of the given years (23.8%, 25.6%, 23.4%, 20.6%, 17.5%). It was observed that the most common mistakes dropped gradually, except in the fall semester of the academic year 2009-2010.

The most common mistakes in IV accesss

The students' mistakes in the practice of IV access were distributed as follows: in the same order of the given years (24.4%, 23.5%, 42.8%, 21.2% and 17.2%), one of the most common mistakes was not following the principles of asepsis. It was verified that this mistake gradually dropped, except in the fall semester of the academic year 2009-2010. Another common mistake was to forget to untie the tourniquet, again in the same order of the given years (19.6%, 20.6%, 14.3%, 21.2% and 22.8%).

Table 2 presents the comparison of the mean NSLE scores between the fall semesters of the years 2005-2006 and 2006-2007, when the NSL studies were not scenario-based, and those in the fall semesters of the years 2007-2012, when the NSL studies were scenario-based. 

**Table 2:** Distribution of average nursing skills laboratory exam marks of second year nursing students. İzmir, Turkey, 2005-2012

Academic Year	n	NSLE* X**^†^** SD**^‡^**		ANOVA F-statistic	p-Value
2005-2006	60	77.56	11.84	8.728	p=0.000
2006-2007	72	80.20	10.28		
2007-2008	82	83.54	8.69		
2008-2009	64	83.06	6.21		
2009-2010	90	81.41	6.55		
2010-2011	105	85.39	5.69		
2011-2012	132	84.01	6.38				

* Nursing skills laboratory exam

† Mean

‡ Standard deviation

The students' mean NSLE scores on the NSL studies that were not scenario-based were lower than their mean scores on the scenario-based studies, except in the fall semester of the academic year2009-2010. The results of the ANOVA test indicated that there were significant differences between the groups (F=8.728, p=0.000). Further analysis (Tukey's HSD test) conducted to identify the source of the differences in the NSLE mean scores revealed significant differences between the fall semesters of the years 2005 and 2007 (p=0.000), 2005 and 2008 (p=0.002), 2005 and 2010 (p=0.000) and 2005 and 2011 (p= 0.000). The mean NSLE score in the fall semester of the year 2005-2006 was lower than in the other years (X=77.56±11.84). There were significant differences between the mean NSLE scores in the fall semesters of 2006, 2010 (p=0.000) and 2011 (p=0.017). The mean NSLE score in the fall semester of the academic year 2006-2007 (X=80.20±10.28) was lower than that of the other years. There was a significant difference between the mean NSLE scores in the fall semesters of the years 2010 and 2009 (p=0.008), and there was a drop in the mean score on the examination done in 2009 (X=81.42±6.55).

### Feedback received from the students and instructors

According to written and verbal feedback received from the students and instructors at the end of each semester, students said that scenario-based skills training helped their learning and made their knowledge permanent, which made it easier to remember on examinations, enabled them to have fun during laboratory studies. They felt readier since they had seen the problems they would encounter in the clinic. The instructors said that the scenarios made students curious, which facilitated their learning and prevented the skills training from being mechanical. 

## Discussion

An analysis of the students' most common mistakes revealed that the most common was not following the principles of asepsis for all three skills (IM and SC injection, IV access) in the first year of the scenario-based training. Mistakes such as forgetting to tell patients to take deep breaths during the injection or forgetting to untie the tourniquet after the IV process can be explained by the fact that the mannequin the procedure was applied on did not give the reactions or response expected from a real human being, along with exam anxiety. In a study, 65% of the participants described OSCE as stressful^14^. In this study, the participants stated in their written feedback after the examination that the NSLE was very stressful and that that was why they forgot the steps of the skill. The scenarios that were included in the following years to reduce these mistakes were effective in making students pay more attention, particularly to asepsis principles, reducing their mistakes. These results were consistent with the increase in students' NSLE achievement scores (F=8.728 p=0.000). The students' NSLE achievement scores increased gradually, except in the fall semester of the academic year 2009-2010. Although the mean NSLE score in the fall semester of the year 2011-2012 decreased slightly after the year 2010-2011, the difference between them was not statistically significant (p=0.833). In studies on the effectiveness of the scenario-based simulation system and its effect on the competencies of nursing students, Alinier, Hunt, Gordon and Harwood found that OSCE 2 scores were higher in the experimental group that received scenario-based training than in the control group^23^. A study was conducted using the Laerdal SimMan Universal Patient Simulator to determine the influence of realistic scenario-based simulation on nursing students' competence and confidence, and it was found that the experimental group had a greater improvement in skill performance than the control group^21^. Another study found that using simulated scenarios was an effective tool to evaluate clinical performance and to make a distinction between the students with high and low performances^24^. It was reported that the students who received simulation training, including scenarios and debriefing in basic life support training, had higher mean scores and had positive opinions about the use of simulation in skills training^4^.

It was determined that, as of the fall semester of 2007-2008, when scenario-based skills training was put into practice, the students' mistakes in NSLE gradually decreased, except in the fall semester of the academic year 2009-2010. There was a decrease in the students' mean NSLE scores in the fall semester of the academic year 2009-2010. According to the written feedback of the instructors, the reason for this was that students neither filled out the rationale section in the control lists nor read the scenarios to solve the problem before coming to the NSL studies. Thus, there was not enough discussion about the rationales and scenarios in the NSL. In their feedback, the students indicated that they preferred to be informed by the instructors instead of coming to the laboratory without any preparation. According to a study by Mete and Uysal, which was conducted in the same school, the instructor gave the lowest scores to the items "writing rationales, discussing rationales and scenario and peer contribution" in the evaluation of the 2009-2010 fall semester laboratory studies^22^. 

The positive feedback provided by the students and instructors at the end of each semester concerning the scenario-based skills training was similar to the results of a study in the literature^25^. Their study reported that the scenario-based simulation method in the fundamental nursing skills laboratory attracted the students' attention and was valuable for clinical experiences. In a study, students stated in their verbal feedback that they were satisfied with the laboratory study^11^. The students' and instructors' feedback indicated that they found the use of the simulation method in nursing education beneficial and suggested that it was used in training. Mete and Uysal analyzed the students' and instructors' feedback in the year 2006-2007, when the scenario-based training was not delivered, and in the first year when it was delivered (2007-2008), and found that both students and instructors had positive opinions about the scenario-based training^22^.

### Study limitation

This study was developed with the second-year students at a nursing school. The study could be developed to include all years at the schools. In addition, the study could include other nursing schools and different training methods.

## Conclusion

Nowadays, advanced technology makes it possible to commonly use high-fidelity simulators. However, the use of low-fidelity simulation in the instruction of non-complex nursing skills cannot be disregarded. The results of this study support the argument that scenario-based skills training in NSL is beneficial for students. This method received a positive response from both students and instructors. The students stated that the scenarios facilitated learning and remembering, showing that this method was beneficial. In conclusion, this method can be used in addition to the other simulation methods in skills training.
